# Abl Tyrosine Kinase Regulates Hepatitis C Virus Entry

**DOI:** 10.3389/fmicb.2017.01129

**Published:** 2017-06-19

**Authors:** Saehong Min, Yun-Sook Lim, Dongjo Shin, Chorong Park, Jae-Bong Park, Seungtaek Kim, Marc P. Windisch, Soon B. Hwang

**Affiliations:** ^1^Department of Biomedical Gerontology, Graduate School of Hallym UniversityChuncheon, South Korea; ^2^National Research Laboratory of Hepatitis C Virus and Ilsong Institute of Life Science, Hallym UniversityAnyang, South Korea; ^3^Hepatitis Research Laboratory, Institut Pasteur KoreaSeongnam, South Korea; ^4^Department of Biochemistry, College of Medicine, Hallym UniversityChuncheon, South Korea; ^5^Institute of Gastroenterology, Yonsei University College of MedicineSeoul, South Korea

**Keywords:** hepatitis C virus, Abl tyrosine kinase, host factor, HCV entry, tyrosine kinase- inhibitor, viral propagation

## Abstract

Abl is a central regulator of multiple cellular processes controlling actin dynamics, proliferation, and differentiation. Here, we showed that knockdown of Abl impaired hepatitis C virus (HCV) propagation. Treatment of Abl tyrosine kinase-specific inhibitor, imatinib and dasatinib, also significantly decreased HCV RNA and protein levels in HCV-infected cells. We showed that both imatinib and dasatinib selectively inhibited HCV infection at the entry step of HCV life cycle, suggesting that Abl kinase activity may be necessary for HCV entry. Using HCV pseudoparticle infection assays, we verified that Abl is required for viral entry. By employing transferrin uptake and immunofluorescence assays, we further demonstrated that Abl was involved in HCV entry at a clathrin-mediated endocytosis step. These data suggest that Abl may represent a novel host factor for HCV entry.

## Introduction

Hepatitis C virus causes both acute and persistent infections and often leads to liver cirrhosis and hepatocellular carcinoma (Saito et al., 1990). HCV is an enveloped RNA virus that belongs to the genus *Hepacivirus* within the family *Flaviviridae* (Giannini and Brechot, 2003). HCV genome consists of 9,600 nucleotides and encodes a single polyprotein precursor of more than 3,000 amino acids. This polyprotein is processed cotranslationally and post-translationally by viral and host cellular proteases into 10 functional proteins, including three structural (core, E1, and E2), the p7 viroporin, and the six non-structural proteins (NS2, NS3, NS4A, NS4B, NS5A, and NS5B) (Lindenbach and Rice, 2005). Although HCV is a highly prevalent pathogen, a vaccine is not available yet. Recently approved DAAs, including boceprevir, telaprevir, sofosbuvir, and simeprevir are successful in treatment of certain HCV patients. However, therapy failure caused by the emergence of DAA-resistance associated variants still remains to be improved (Carlson et al., 2013). An alternative way to develop HCV therapy is HTAs. Targeting host protein has a high genetic barrier to resistance and the potential for pangenotypic antiviral activity. HCV relies on host cellular machinery for all steps of its life cycle. Blocking any step of the virus life cycle results in an efficient blockade of viral production, and thereby it could be a potential target for HCV therapy.

Hepatitis C virus enters the host cells by receptor-mediated endocytosis (Blanchard et al., 2006). HCV entry is a multistep process that requires sequential interactions between viral proteins and host factors, including CD81, scavenger receptor class B type I (SR-BI), claudin-1 (CLDN1), occludin (OCLN), epidermal growth factor receptor (EGFR), and low-density lipoprotein (LDL) receptor (Monazahian et al., 1999; Scarselli et al., 2002; Bartosch et al., 2003; Brazzoli et al., 2008; Liu et al., 2009; Lupberger et al., 2011; Farquhar et al., 2012; Lindenbach and Rice, 2013). Cellular kinases play a key role in the movement and uptake of virus (Zona et al., 2013). Abl is a member of the non-receptor tyrosine kinase family which transduces diverse extra and intracellular signals to regulate multiple cellular processes controlling actin reorganization, cell proliferation, and differentiation (Reddy et al., 1983; Colicelli, 2010). Recent studies showed that some pathogens exploit Abl kinase signaling to rearrange F-actin cytoskeleton and trigger phosphorylation of bacterial and viral effector proteins. Abl signaling has been reported to be involved in the diverse microbial invasion and release from host cells, actin-tail formation, pedestal formation, and host cell scattering (Woodring et al., 2003; Backert et al., 2008). In the present study, we demonstrated that genetic knockdown or pharmacological inhibition of Abl impaired HCV propagation. We further showed that Abl tyrosine kinase-specific inhibitors, imatinib and dasatinib, selectively inhibited the entry step of the HCV life cycle. Moreover, we showed that Abl was specifically involved in clathrin-mediated endocytosis. These data suggest that Abl is a host factor required for HCV entry, and thereby it may be a potential target for the treatment of HCV infection.

## Materials and Methods

### Plasmid Construction

To express recombinant HCV soluble E2 (sE2) (aa 384–661), C-terminal transmembrane region of E1 (aa 364–383) was included to increase protein expression level. Therefore, cDNA corresponding to the aa 364–661 was amplified by PCR using pH77S.3/Gluc2A as a template with following primer set (sense, 5′-ATG GAT CCA TGG TGG GGA ACT GGG CGA-3′; anti-sense, 5′-ATG AAT TCC TCG GAC CTG TCC CTG TCT-3′). PCR products were inserted into the BamHI and EcoRI sites of the plasmid pEF6A-Myc-6XHis (Invitrogen). The resultant plasmid is named as pEF6A-sE2-Myc-6XHis. To construct Abl expression plasmid, cDNA was synthesized from total RNAs isolated from Huh7.5 cells by using a cDNA synthesis kit (Toyobo) according to the manufacturer’s instruction. Full-length Abl was amplified by a primer set (sense, 5′-CGA AGC TTA TGT TGG AGA TCT GCC TG-3′; antisense, 5′-CCT CTA GAC TAC CTC TGC ACT ATG TC-3′) and PCR products were inserted into the HindIII and XbaI sites of p3XFlag-CMV10 vector (Sigma). A small interfering RNA (siRNA)-resistant Abl mutant was constructed by introducing two mutations at the siRNA binding site using a primer set (5′-TGT TGA TCC TGT AGT GAT ACA CCC TCC CTT CGT GC-3′; 5′-GCT GAG ATA CGA AGG GAG GGT GTA TCA CTA CAG GAT CAA CA-3′). Huh7.5 cells were transiently transfected with 2 mg of p3XFlag-Abl or p3XFlag-Abl-SR plasmid using polyethyleneimine (Sigma). PFK-Jc1 (Pietschmann et al., 2006) was kindly provided by Dr. Ralf Bartenschlager (University of Heidelberg).

### Cell Culture

All cell lines were grown in Dulbecco’s modified Eagles’ medium (DMEM) supplemented with 10% fetal bovine serum, 1% penicillin/streptomycin in 5% CO_2_ at 37°C. Huh7 cells harboring HCV subgenomic replicon derived from genotype 1b were grown as we reported previously (Nguyen et al., 2014). Primary human hepatocytes were cultured in hepatocyte medium (ScienCell Research Laboratories) supplemented with 5% fetal bovine serum, 1% hepatocyte growth factor, and 1% penicillin/streptomycin in 5% CO_2_ at 37°C.

### Chemicals and Antibodies

Both imatinib and dasatinib, tyrosine kinase-specific inhibitors, were purchased from Axon Medchem. Bafilomycin A1 and Texas Red conjugated transferrin were purchased from Sigma–Aldrich. Sofosbuvir (PSI-7977) was purchased from MedChem Express. Antibodies were purchased from the following sources: rabbit anti-Abl kinase antibodies, anti-phosphor-CRKL (Y207) antibodies, and anti-EGFR antibodies from Cell Signaling; anti-clathrin heavy chain antibodies, and anti-CD81 neutralizing antibodies from BD bioscience; anti-β-actin antibodies from Sigma–Aldrich. Rabbit anti-NS5A, anti-NS3, and anti-core antibodies have been described elsewhere (Ngo et al., 2013). HCV E2 antibody was a gift from Dr. Jean Dubuisson (Institut Pasteur de Lille). Transfection reagents used were either Lipofectamine 2000 (Invitrogen, Carlsbad, CA, United States) or polyethyleneimine (Sigma–Aldrich).

### Generation of HCV

Infectious HCVs were generated as we previously described (Park et al., 2015) with minor modifications. Briefly, the pFK-Jc1 plasmid was linearized at the 3′ end of the Jc1 genome by MluI digestion. The pJFH1 E2p7-5A/5B-GFP plasmid (Lee et al., 2017) and pH77D plasmid (Yamane et al., 2014) were linearized at the 3′ end of the HCV genome by XbaI digestion. Plasmids were purified by phenol–chloroform extraction. RNA transcripts were generated using T7 RiboMAX^TM^ Express Large Scale RNA Production System (Promega). The generated HCV RNA was further purified by RiboEX LS (GeneAll) according to the manufacturer’s protocol. Ten micrograms of *in vitro* transcribed genomic Jc1 RNA was mixed with 6 × 10^6^ Huh7.5 cells in 0.4-cm gap cuvette and electroporated at 270 V and 950 μF using a Bio-Rad GenePulser II electroporator. Cells were gently transferred to complete medium (low glucose DMEM containing 10% FBS, 1% penicillin/streptomycin, 2 mM L-glutamine, 1 mM NEAA, and 10 mM HEPES) and plated on a 150-mm dish. At 24 h later, the medium was replaced with the fresh complete medium to remove dead cells. At 4 days after electroporation, the culture medium was collected, filtered through a 0.45-mm syringe-top filter unit (Milipore) and kept as a virus stock.

### Preparation of HCV Pseudoparticles

HEK293T cells were transfected with human immunodeficiency virus type 1 (HIV 1) Gag-pol packaging plasmid, firefly luciferase reporter plasmid, and plasmid expressing HCV envelope protein derived from either genotype 1a (H77) or 2a (JFH1), or pVSV-G (Clontech, Mountain View, CA, United States) expressing the vesicular stomatitis virus (VSV) envelope glycoprotein G. Transfections were performed for 6 h using Lipofectamine 2000 as instructed by the manufacturer (Invitrogen). At 72 h after transfection, media containing HCV pseudoparticles (HCVpp) or VSV pseudoparticles (VSVpp) were collected by centrifugation at 1500 rpm and further clarified by passage through a 0.45 μm filters (Millipore, Bedford, MA, United States). HCVpp and VSVpp were further purified by using Amicon ultra-15 centrifugal filter devices with a 10-kDa cutoff (Millipore), and stored at -70°C as a viral stock.

### Production and Purification of sE2

HEK293T cells were transiently transfected with pEF6A-sE2-Myc-6XHis plasmid by using polyethyleneimine (Sigma–Aldrich) for 4 h according to the manufacturer’s instructions. The culture media were replaced with DMEM supplemented with 2% fetal bovine serum. The culture media containing sE2 were harvested at 48 h after transfection and clarified by centrifugation at 2000 rpm, and then concentrated by Microcon (Millipore). To quantitate protein concentration of H77 sE2, media containing sE2 were incubated with Ni-NTA agarose beads (Quiagen) for 1 h at 4°C. The sE2 was purified using an elution buffer 50 mM NaH_2_PO_4_, 300 mM NaCl, 250 mM imidazole, 0.1% Tween 20 (pH 8.0), 1 mM PMSF, and protease inhibitor cocktail on Ni-NTA agarose columns (Quiagen) according to the manufacturer’s instructions. The purified H77 sE2 was dialyzed by Slide-A-Lyzer G2 Dialysis Cassettes, 10K MWCO (Thermo Scientific). The protein concentration of H77 sE2 was determined by the Bradford assay (Bio-Rad).

### Quantitative Real-Time PCR Analysis

Total RNAs were isolated using RIBO EX (Gene All) according to the manufacturer’s instructions. cDNA was synthesized using the Reverse Tra Ace-α-cDNA synthesis kit (TOYOBO). Quantification of RNA was carried out using CFX connect TM real-time PCR detection system under the following conditions: 3 min at 95°C, followed by 40 cycles of 95°C for 10 s, 56°C for 10 s, and 72°C for 30 s. Seventy one cycles of 5 s, with 0.5°C temperature increments from 60 to 95°C, were used for the melting curves. The primer sequences used were as follows: HCV 5′ UTR of genotype 2-specific primers (forward, TGA GTG TCG TAC AGC CTC CA; reverse, ACG CTA CTC GGC TAG CAG TC); HCV 5′ UTR of genotype 1-specific primers (forward, TCT GCG GAA CCG AGT A; reverse, TCA GGC AGT ACC ACA AGG C), β-actin-specific primers (forward, GAC TTC CTG TAA CAA CGC ATC TCA TA; reverse, TGA AAA GCT CCG GGT CTT AGG), GAPDH-specific primers (forward, CGC TCT GTG CTC CTC CTG TTC; reverse, CGC CCA ATA CGA CCA AAT CCG) and Abl-specific primers (forward, TGA AAA GCT CCG GGT CTT AGG; reverse, TTG ACT GGC GTG ATG TAG TTG).

### RNA Interference

siRNAs targeting Abl (#1, 5′-GAA GGG AGG GUG UAC CAU UUU-3′; #2, 5′-AAC GGC UGA UGU GGA CUG UCU UU-3′) (Xu et al., 2010; Murray et al., 2014), CD81 (5′-AUC UGG AGC UGG GAG ACA AUU-3′) (Brazzoli et al., 2008), 5′ NTR of Jc1 (5′-CCU CAA AGA AAA ACC AAA CUU-3′), and the universal negative control siRNA were purchased from Bioneer. siRNA transfection was performed using the Lipofectamine RNAiMax reagent (Invitrogen, Carlsbad, CA, United States) according to the manufacturer’s instructions.

### Immunofluorescence Assay

Huh7.5 cells cultured on glass slides were treated with either DMSO or imatinib for 2 h. Huh7.5 cells treated with negative control siRNA or Abl-specific siRNA were cultured on glass slides for 48 h. Cells were then incubated with Jc1 (MOI of 5) for 30 min. Cells were washed twice with cold PBS and then fixed with 4% paraformaldehyde. After three washes with PBS, fixed cells were blocked with 1% BSA in PBS for 1 h at room temperature. The cells were then incubated with mouse anti-clathrin heavy chain monoclonal antibody overnight at 4°C. After being washed three times with PBS, cells were further incubated with tetramethylrhodamine isothiocyanate (TRITC)-conjugated donkey anti-mouse secondary antibody (Jackson Immunoresearch Laboratories) for 1 h at room temperature. After two washes with PBS, cells were analyzed using the Zeiss LSM 700 laser confocal microscopy system (Carl Zeiss, Inc., Thornwood, NY, United States).

### WST Assay

Huh7.5 cells were treated with water-soluble tetrazolium salt (WST) reagent (Dail Lab). At 2 h after WST treatment, the absorbance was measure at 450 and 650 nm as reference wavelength using a VersaMax microplate reader (Molecular Devices).

### TCID50 Assay

A 50% tissue culture infectious dose (TCID_50_) assay was performed as we reported previously (Pham et al., 2017).

### Time-of-Addition Assays

Approximately, 3 × 10^3^ Huh7.5 cells/well were seeded on 384-well plates. At 16 h after plating, cells were inoculated with JFH1 E2p7-5A/5B-GFP virus (MOI of 5) for 2 h at 4°C for binding, and then washed twice with cold media to remove unbound viruses. Temperature was shifted to 37°C and then cells were incubated with either DMSO, imatinib (10 μM), bafilomycin A1 (10 nM), sofosbuvir (2 μM), or CD81-neutralizing antibodies (2 μg/ml) for the indicated time points. At 72 h post-infection, HCV protein expression levels were quantified by measuring GFP positive cells.

### Transferrin Uptake Assay

To investigate clathrin-mediated endocytosis, 5 × 10^3^ Huh7.5 cells/well were seeded on 384-well plates for 16 h. Cells were washed and pre-treated with either DMSO or various doses of imatinib or chlorpromazine in serum-free DMEM. Cells were then incubated with 10 μg/ml Texas Red conjugated transferrin in serum-free DMEM supplemented with 1% BSA for 1 h at 4°C. Temperature was then shifted to 37°C for 20 min to allow cells to uptake transferrin. Cells were washed with PBS twice, and then fixed in 4% paraformaldehyde. Transferrin uptake was quantitated by measuring fluorescence intensity of internalized transferrin using ImageJ software.

### Membrane Fluidity Assay

Huh7.5 cells were seeded on 96-well plates for 16 h, and then treated with either DMSO or various doses of imatinib or trifluoperazine for 2 h at 37°C. Cells were incubated with dye for 20 min at 25°C and then membrane fluidity was determined as reported previously (Parasassi et al., 1990).

### Statistical Analysis

Data are presented as means ± standard deviations (SD) (*n* = 3 or as indicated). Student’s *t*-test was used for statistical analysis. The asterisks indicate significant differences (^∗^*P* < 0.05; ^∗∗^*P* < 0.01; ^∗∗∗^*P* < 0.001).

## Results

### Abl Is Required for HCV Propagation

**FIGURE 1:**
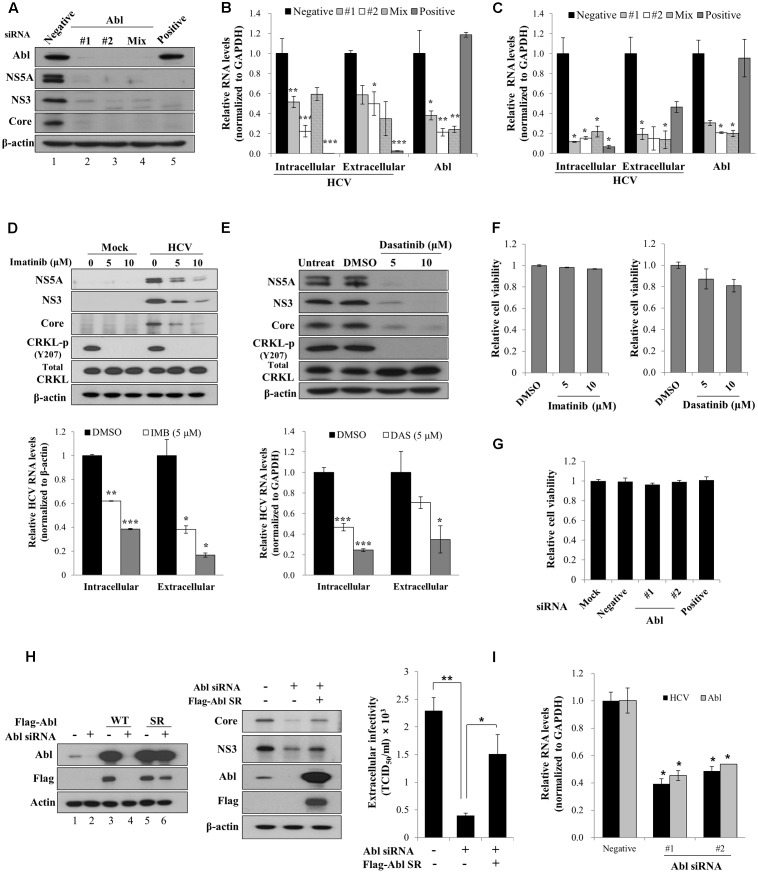
Abl is required for HCV propagation. **(A)** Huh7.5 cells were transfected with 10 nM concentration of the indicated siRNAs. At 48 h after transfection, cells were then infected with Jc1 for 4 h using an MOI of 0.5. Total cell lysates harvested at 48 h post-infection were immunoblotted with the indicated antibodies. Negative denotes universal negative control siRNA. Positive indicates HCV-specific siRNA targeting the 5′ NTR of Jc1. **(B)** Huh7.5 cells were treated as described in **(A)** and then RNA levels of both HCV and Abl were quantified by qRT-PCR. Experiments were carried out in triplicate. The asterisks indicate significant differences (^∗^*P* < 0.05; ^∗∗^*P* < 0.01, ^∗∗∗^*P* < 0.001). Error bars indicate the standard deviations of the means. **(C)** Huh7.5 cells were transfected with 10 nM concentration of the indicated siRNAs. At 48 h after transfection, cells were then infected with H77D for 4 h. At 48 h post-infection, RNA levels of both HCV and Abl were analyzed by qRT-PCR. **(D)** (Upper panel) Huh7.5 cells pretreated with imatinib were either mock-infected or infected with Jc1 for 4 h. Cells were further incubated with either DMSO (vehicle) or two different doses of imatinib for 48 h. Total cell lysates were immunoblotted with the indicated antibodies. (Lower panel) Both intracellular and extracellular HCV RNA levels were determined by qRT-PCR. **(E)** (Upper panel) Huh7.5 cells were pretreated with either DMSO or two different doses of dasatinib and then infected with Jc1 for 4 h. Cells were further incubated with either DMSO or dasatinib for 48 h. Total cell lysates were immunoblotted with the indicated antibodies. (Lower panel) Both intracellular and extracellular HCV RNA levels were determined by qRT-PCR. Experiments were performed in triplicate. The asterisks indicate significant differences (^∗^*P* < 0.05; ^∗∗^*P* < 0.01, ^∗∗∗^*P* < 0.001). **(F)** Huh7.5 cells were treated with either DMSO or two different doses of imatinib (left panel) or dasatinib (right panel). Forty-eight hours after inhibitor treatment, cell viability was determined by a WST assay. **(G)** Huh7.5 cells were transfected with 20 nM of the indicated siRNAs. At 72 h after transfection, cell viability was determined by a WST assay. **(H)** (Left panel) Huh7.5 cells were transfected with p3XFlag-Abl wild-type (WT) or p3XFlag-Abl siRNA-resistant (SR) expression plasmid. At 24 h after transfection, total cell lysates were immunoblotted with the indicated antibodies. (Middle panel) Huh7.5 cells were transfected with either negative siRNA or Abl-specific siRNA. At 24 h transfection, cells were further transfected with Flag-tagged Abl SR plasmid for 24 h and then infected with Jc1 for 4 h. Cell lysates harvested at 2 days post-infection were immunoblotted with the indicated antibodies. (Right panel) Naïve Huh7.5 cells were inoculated with the supernatant harvested from experiment described in the middle panel. At 2 days post-infection, cells were fixed with methanol and HCV infectivity was determined by TCID50 assay. **(I)** Primary human hepatocytes were transfected with negative siRNA or Abl-specific siRNAs. At 2 days after transfection, cells were infected with Jc1 for 4 h. Cells were harvested at 2 days post-infection and then intracellular RNA levels of HCV and Abl were determined by qRT-PCR.

It has been previously reported that Abl tyrosine kinase is implicated in viral pathogenesis (Coyne and Bergelson, 2006; Sato et al., 2012). To investigate the functional involvement of Abl in HCV propagation, Huh7.5 cells transfected with siRNAs were infected with Jc1 (genotype 2a). At 2 days post-infection, total cellular lysates were immunoblotted with the indicated antibodies. The positive siRNA targeting 5′ UTR of HCV completely blocked HCV protein expression (**Figure 1A**, lane 5). Similarly, siRNA mediated knockdown of Abl led to strong inhibition of both structural and non-structural HCV protein expressions (**Figure 1A**, lanes 2 and **3**). We further showed that both intracellular and extracellular HCV RNA levels were also significantly decreased in Abl knockdown cells (**Figure 1B**). To further verify the role of Abl in HCV propagation, Huh7.5 cells transfected with siRNAs were infected with HCV H77D (genotype 1a). As shown in **Figure 1C**, knockdown of Abl impaired both intracellular and extracellular HCV RNA expression levels in HCV H77D-infected cells, further confirming that Abl is involved in HCV propagation. It has been previously reported that both imatinib and dasatinib specifically inhibit Abl kinase (Weisberg et al., 2007; Backert et al., 2008). To demonstrate whether Abl kinase activity was required for HCV propagation, Huh7.5 cells treated with imatinib were either mock-infected or infected with Jc1 and then total cell lysates were immunoblotted with the indicated antibodies. We demonstrated that imatinib markedly inhibited HCV propagation at the levels of protein (**Figure 1D**, upper panel) and HCV RNA (**Figure 1D**, lower panel) in a dose-dependent manner. Consistently, HCV propagation was impaired in dasatinib-treated Huh7.5 cells (**Figure 1E**). One intriguing question was that total CRKL protein levels were slightly increased in both imatinib- and dasatinib-treated Huh7.5 cells. Nevertheless, both Abl inhibitors efficiently blocked CRKL phosphorylation. We also showed that both imatinib and dasatinib displayed no cell toxicity (**Figure 1F**). To further evaluate the cytotoxicity of siRNAs, Huh7.5 cells were transfected with the indicated siRNAs and cell viability was determined by a WST assay. As shown in **Figure 1G**, Abl siRNAs exerted no cytotoxicity to Huh7.5 cells. To rule out the off-target effect of Abl siRNA, we generated an siRNA-resistant Abl mutant. We first demonstrated that Abl protein expression was not impaired in siRNA-resistant Abl mutant-transfected cells (**Figure 1H**, lane 6 in left panel). Using this mutant, we showed that exogenous expression of the siRNA-resistant Abl mutant rescued the HCV protein expression level. These data show that Abl is specifically required for HCV propagation. Using siRNA-resistant Abl mutant, we further verified that Abl was involved in HCV propagation by TCID50 assay (**Figure 1H**, right panel). To finally prove the role of Abl in primary cells, primary human hepatocytes transfected with Abl siRNAs were infected with Jc1. As shown in **Figure 1I**, intracellular HCV RNA levels are significantly reduced in Abl depleted cells, further confirming that Abl tyrosine kinase activity is required for HCV propagation.

### Abl Is Not Involved in the Replication Step of the HCV Life Cycle

**FIGURE 2:**
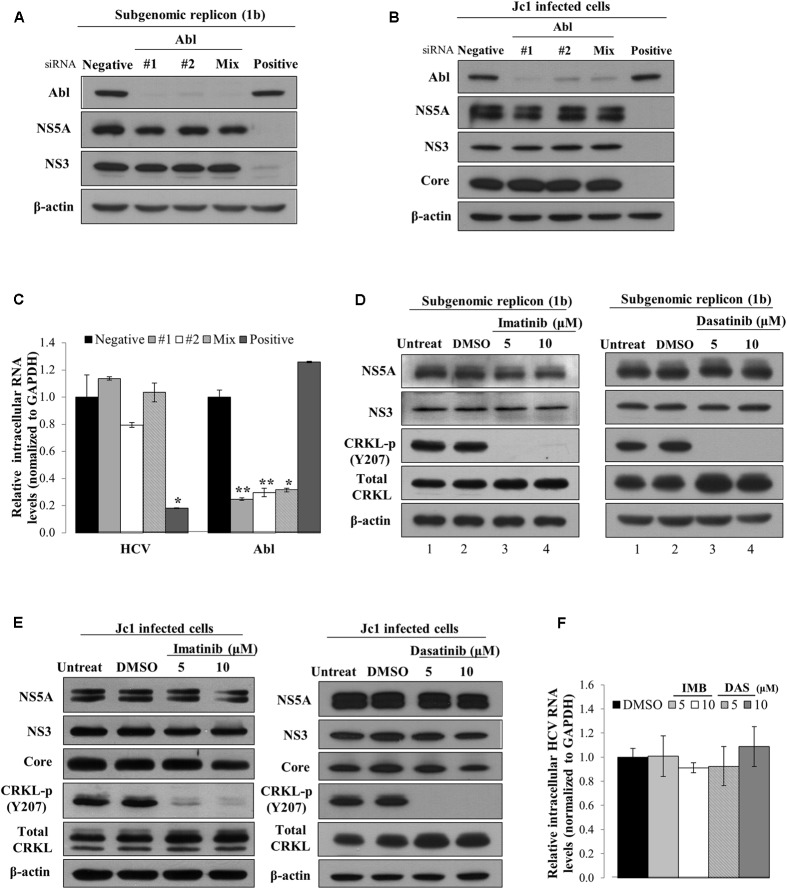
Abl is not involved in the replication stage of the HCV life cycle. **(A)** Huh7 cells harboring HCV subgenomic replicon derived from genotype 1b were transfected with the indicated siRNAs. At 3 days after transfection, total cell lysates were immunoblotted with the indicated antibodies. **(B)** Huh7.5 cells were infected with Jc1 for 4 h. At 48 h post-infection, cells were transfected with the indicated siRNAs. Total cell lysates harvested at 2 days after transfection were immunoblotted with the indicated antibodies. **(C)** Huh7.5 cells were treated as described in **(B)** and then intracellular RNA levels of both HCV and Abl were quantified by qRT-PCR. The asterisks indicate significant differences (^∗^*P* < 0.05; ^∗∗^*P* < 0.01). **(D)** Huh7 cells harboring HCV subgenomic replicon (genotype 1b) were either left untreated or treated with DMSO, or the indicated concentrations of imatinib (left panel) or dasatinib (right panel). At 72 h after inhibitor treatments, total cell lysates were immunoblotted with the indicated antibodies. **(E)** Huh7.5 cells were infected with Jc1 for 4 h. At 48 h post-infection, cells were left untreated or treated with either DMSO or the indicated concentrations of imatinib (left panel) or dasatinib (right panel). Forty-eight hours after inhibitor treatments, total cell lysates were immunoblotted with the indicated antibodies. **(F)** Huh7.5 cells were treated as described in **(E)** and then intracellular HCV RNA levels were quantified by qRT-PCR.

To investigate which steps of the HCV life cycle were required for Abl, Huh7 cells harboring HCV subgenomic replicon were transfected with the indicated siRNAs. At 72 h after transfection, total cell lysates were immunoblotted with the indicated antibodies. As shown in **Figure 2A**, silencing of Abl exerted no effect on protein expression levels in subgenomic replicon cells. To further confirm the effect of Abl on HCV replication, Huh7.5 cells were infected with Jc1. At 48 h post-infection, Huh7.5 cells were transfected with the indicated siRNAs and then total cell lysates were immunoblotted with the indicated antibodies. We showed that knockdown of Abl displayed no effect on HCV protein expression levels in Jc1-infected cells (**Figure 2B**). Consistently, intracellular HCV RNA levels were not altered in Abl knockdown cells (**Figure 2C**). Next, we explored the effect of chemical inhibition of Abl kinase on HCV replication. Huh7 cells harboring HCV replicon were either left untreated or treated with DMSO, or the indicated amounts of imatinib. At 72 h after inhibitor treatment, total cell lysates were immunoblotted with the indicated antibodies. CRKL is a well-known downstream effector of Abl (Ten Hoeve et al., 1994). As shown in **Figure 2D**, phosphorylation levels of CRKL were efficiently suppressed by imatinib (lanes 3 and 4 in the left panel). However, Abl kinase inhibitor displayed no effect on HCV protein expression levels in replicon cells. Consistently, dasatinib had no effect on HCV protein expressions levels (**Figure 2D**, right panel). To further verify the role of Abl in HCV replication, Huh7.5 cells infected with Jc1 were treated with Abl inhibitors. At 48 h after inhibitor treatments, total cell lysates were immunoblotted with the indicated antibodies. **Figure 2E** shows that Abl kinase activities were dramatically suppressed by either imatinib (left panel) or dasatinib (right panel). Nevertheless, both structural and non-structural HCV protein levels were not affected by Abl kinase inhibitor. Consistently, Abl inhibitors displayed no effects on intracellular HCV RNA levels (**Figure 2F**). Collectively, neither genetic knockdown nor chemical inhibition of Abl kinase displayed an inhibitory effect on HCV replication. These data suggest that Abl may be involved in other steps of the HCV life cycle.

### Abl Kinase Is Required for the Entry Step of HCV Life Cycle

**FIGURE 3:**
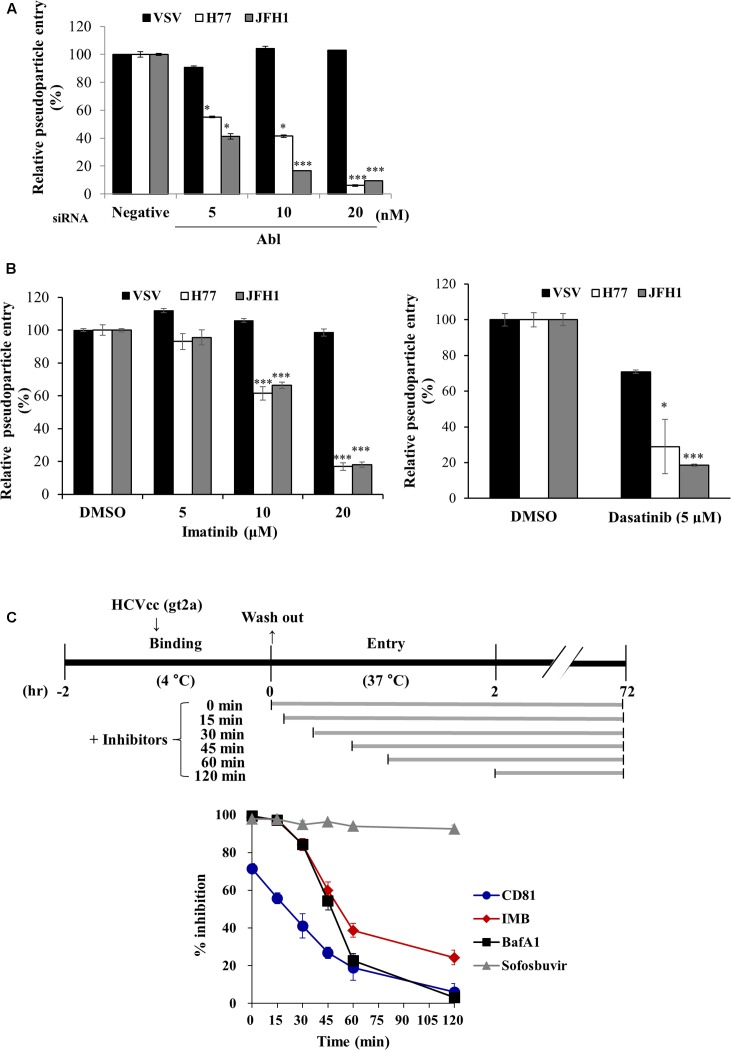
Abl is required for HCV entry. **(A)** Huh7.5 cells were transfected with the indicated siRNAs for 48 h and then infected with either VSVpp or HCVpp derived from genotype 1a (H77) and 2a (JFH1) for 6 h. At 72 h post-infection, cells were harvested and viral entry was determined by luciferase activity. Values are presented as means ± SD (*n* = 3). The asterisks indicate significant differences (^∗^*P* < 0.05; ^∗∗∗^*P* < 0.001) from the value for the negative control. **(B)** Huh7.5 cells were treated with DMSO or the indicated concentration of imatinib (left panel) or dasatinib (right panel) for 4 h. Cell were then infected with either VSVpp or HCVpp derived from genotype 1a (H77) and 2a (JFH1) for 6 h. At 72 h post-infection, viral entry was determined by luciferase activity. **(C)** (Upper panel) Schematic illustration of the experimental design. Huh7.5 cells were incubated with GFP-tagged JFH1 (E2p7-5A/5B-GFP; Lee et al., 2017) using an MOI of 5 for binding at 4°C for 2 h. After the cells had been washed in PBS, the temperature was shifted to 37°C to allow cell entry in the absence or presence of inhibitors and neutralizing antibodies of CD81 for the indicated time periods. (Lower panel) At 72 h post-infection, HCV entry was determined by GFP-positive cells. Sofosbuvir was used as a positive control. IMB, imatinib; BafA1, bafilomycin A1.

To further investigate the possible involvement of Abl in HCV life cycle, we employed HCVpp entry assay. Huh7.5 cells transfected with the indicated siRNAs were inoculated with either HCVpp derived from JFH1 or H77 strain, or VSVpp. We demonstrated that silencing of Abl significantly decreased HCVpp entry of both strains but not VSVpp entry (**Figure 3A**). Next, we explored the effect of Abl kinase activity on HCV entry. For this purpose, Huh7.5 cells were treated with DMSO or the indicated concentrations of imatinib or dasatinib. As shown in **Figure 3B**, HCV entry was blocked by either imatinib (left panel) or dasatinib (right panel) in a dose-dependent manner. These data suggest that HCV may exploit cellular Abl for its entry to the target cells and Abl kinase activity is required for entry process. To determine more precisely which step of HCV entry was required for Abl kinase activity, we performed time-of-addition assays as we reported previously (Bush et al., 2014) by using the indicated inhibitors and neutralizing antibodies. Huh7.5 cells were incubated with Jc1 for 2 h at 4°C and then temperature was shifted to 37°C. Cells were then treated with either inhibitors or neutralizing antibodies for the indicated time intervals (**Figure 3C**, upper panel). At 72 h post-infection, HCV entry was determined by measuring GFP positive cells as we reported elsewhere (Lee et al., 2017). Bafilomycin A1, a well-known endosomal inhibitor, blocks HCV entry (Baldick et al., 2010), whereas CD81 is involved in binding step of the HCV infection (Brazzoli et al., 2008). We demonstrated that anti-CD81 antibody exerted a time of half-maximal inhibition of entry (t_50_) of 22.1 ± 4.5 min, whereas t_50_ for bafilomycin A1 was 47.1 ± 2.3 min. Since t_50_ for imatinib was 51.7 ± 2.7 min, the inhibitory effect of imatinib on HCV infection was similar to that of bafilomycin A1 (**Figure 3C**, lower panel). These data clearly indicate that Abl tyrosine kinase is required for the post-binding step of the HCV life cycle.

### HCV Modulates Abl Kinase Activity

**FIGURE 4:**
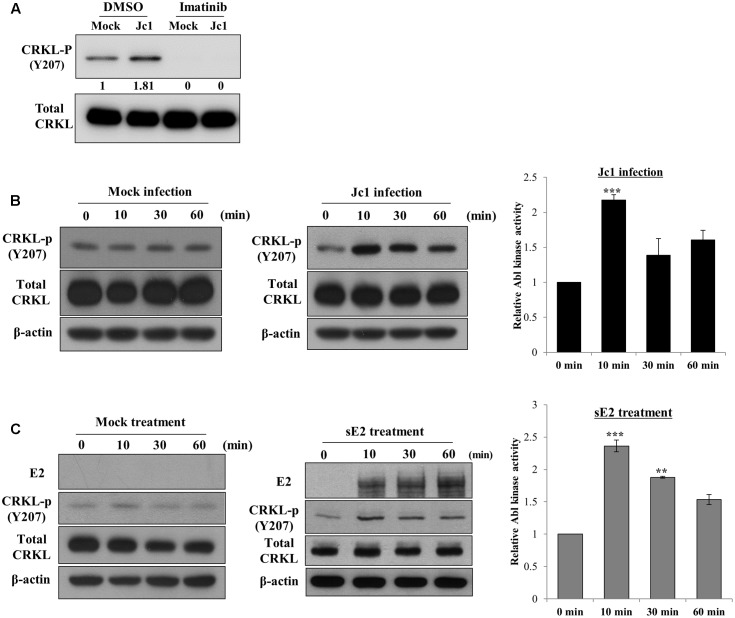
Hepatitis C virus infection activates Abl kinase. **(A)** Huh7.5 cells were serum-starved for 16 h and then treated with either DMSO (vehicle) or 10 μM of imatinib for 2 h. Cells were either mock-infected or infected with Jc1 using an MOI of 5. At 10 min post-infection, total cell lysates were immunoblotted with the indicated antibodies. **(B)** Huh7.5 cells were serum-starved for 16 h and then either mock-infected (left panel) or infected with Jc1 (middle panel). At the indicated time points, total cell lysates were immunoblotted. (Right panel) Abl kinase activities were determined by measuring band intensities of CRKL-p/total CRKL using ImageJ and shown as relative fold from the mock control. **(C)** Huh7.5 cells were serum-starved for 16 h and then either mock-treated (left panel) or treated with 10 μg sE2 derived from H77 (middle panel). Total cell lysates harvested at each time points were immunoblotted with the indicated antibodies. (Right panel) Abl kinase activities were determined by measuring band intensities of CRKL-p/total CRKL using ImageJ and shown as relative fold from the mock control. sE2, soluble HCV E2 protein. The asterisks indicate significant differences (^∗∗^*P* < 0.01; ^∗∗∗^*P* < 0.001).

To investigate whether Abl kinase activity was modulated by HCV infection, Huh7.5 cells were treated with either DMSO or imatinib for 2 h and then infected with Jc1. At 10 min post-infection, total cell lysates were immunoblotted with the indicated antibodies. As shown in **Figure 4A**, phosphorylation level of CRKL was immediately increased by HCV infection and imatinib completely blocked HCV-induced CRKL phosphorylation, indicating that Abl kinase activity was upregulated by HCV infection. To further verify this result, Huh7.5 cells were either mock-infected or infected with Jc1 and then cell lysates harvested at various time points were immunoblotted with the indicated antibodies. As shown in **Figure 4B**, Abl kinase activity was unchanged in mock-infected cells (left panel). However, Abl kinase activity was increased more than twofold immediately after Jc1 infection and then leveled off slowly (**Figure 4B**, middle and right panels). Since Abl kinase was activated at the entry step of the HCV infection, we further examined whether Abl kinase was also activated by HCV E2 protein. For this purpose, Huh7.5 cells were either mock-treated or treated with sE2 derived from H77 and then cell lysates harvested at various time points were immunoblotted with the indicated antibodies. Indeed, Abl kinase activity was markedly increased at 10 min after sE2 treatment (**Figure 4C**, middle and right panels), whereas Abl kinase was not activated in mock-treated cells (left panel). These data suggest that HCV modulates Abl kinase activity via E2 for viral entry into the host cells.

### Abl Is Involved in Clathrin-Mediated Endocytosis of HCV Entry

**FIGURE 5:**
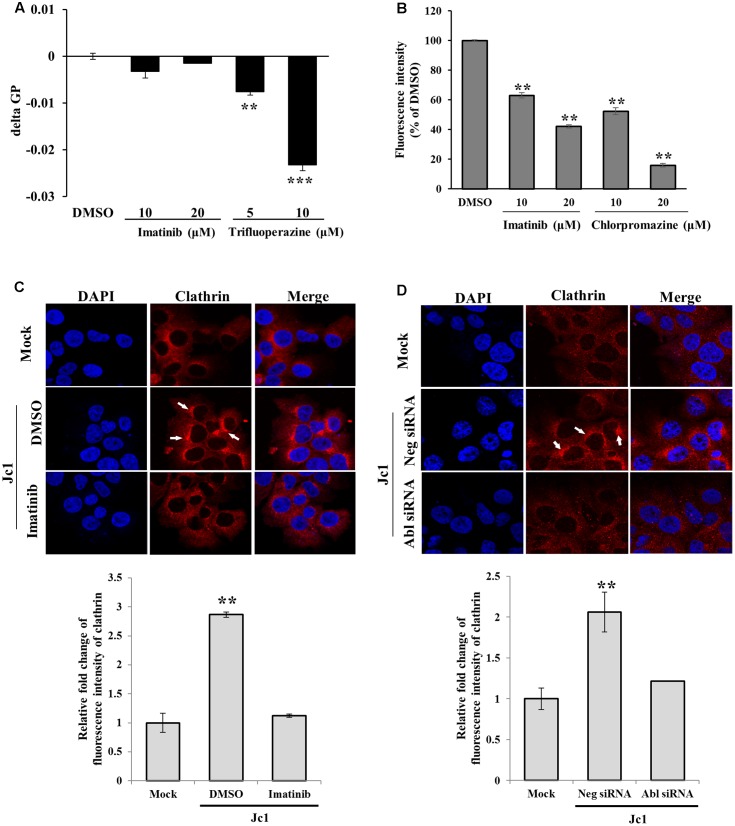
Abl is required for the clathrin-mediated endocytosis. **(A)** Huh7.5 cells were treated with either DMSO or the indicated concentration of imatinib or trifluoperazine for 2 h at 37°C. Cells were further treated with membrane fluidity detection dye for 20 min at 25°C and then wavelength was detected. Generalized polarization (GP) = (*I*405-*I*460)/(*I*405+*I*460), where *I*405 and *I*460 are the fluorescence intensity measured under wavelength 405 and 460, respectively. The asterisks indicate significant differences (^∗∗^*P* < 0.01; ^∗∗∗^*P* < 0.001) from the value for the DMSO control. **(B)** Huh7.5 cells were incubated with serum-free DMEM in the presence of either DMSO or the indicated concentrations of imatinib or chlorpromazine. The cells were incubated with 10 μg/ml Texas Red-conjugated transferrin in serum-free DMEM supplied with 1% BSA for 1 h at 4°C. The cells were fixed in 4% paraformaldehyde. Fluorescence intensity was determined by measuring the internalized transferrin using Image J. **(C)** (Upper panel) Huh7.5 cells were treated with either DMSO or imatinib for 4 h at 37°C and then infected with Jc1 using an MOI of 5. The cells were then fixed in 4% paraformaldehyde and stained with anti-clathrin heavy chain and TRITC antibodies. Cells were counterstained with 4′,6-diamidino-2-phenylindole (DAPI) to label nuclei (blue). (Lower panel) Fluorescence intensity of clathrin was quantified by using ImageJ software. **(D)** (Upper panel) Huh7.5 cells were transfected with either negative siRNA or Abl-specific siRNAs for 48 h and then infected with Jc1 using MOI of 5. At 30 min post-infection, cells were fixed in 4% paraformaldehyde and stained with anti-clathrin heavy chain and TRITC antibodies. Cells were counterstained with DAPI to label nuclei. (Lower panel) Fluorescence intensity of clathrin was quantified by using ImageJ software.

To investigate how Abl was involved in HCV entry, we performed membrane fluidity assay. Trifluoperazine is an inhibitor which abolishes HCV entry at the step of virus-host cell fusion by altering membrane fluidity (Chamoun-Emanuelli et al., 2013). As shown in **Figure 5A**, imatinib displayed no effect on membrane fluidity. On the other hand, membrane fluidity was significantly reduced by trifluoperazine. Since transferrin uptake reflects clathrin-mediated endocytosis, Huh7.5 cells were treated with Texas Red-conjugated transferrin and then transferrin uptake was determined by measuring fluorescence intensity. As shown in **Figure 5B**, transferrin uptake was significantly decreased by imatinib in a dose-dependent manner. Chlorpromazine, a drug that efficiently blocks clathrin-mediated endocytosis, also significantly disturbed transferrin uptake in Huh7.5 cells. To further investigate whether Abl was involved in clathrin-mediated endocytosis of HCV, we performed immunofluorescence assay to detect clathrin in mock-infected or HCV-infected cells. Huh7.5 cells were infected with Jc1 for 30 min and then alteration of clathrin expression was analyzed. As shown in **Figure 5C**, clathrin was actively aggregated at 30 min post-infection in Jc1-infected cells as compared to mock-infected cells. However, imatinib treatment caused a reduction in clathrin aggregation in Jc1-infected cells. To further verify these results, Huh7.5 cells were transfected with either negative control siRNA or Abl-specific siRNA for 48 h and then infected with Jc1. At 30 min post-infection, cells were stained with clathrin antibody. As expected, silencing of Abl displayed no aggregation of clathrin in HCV-infected cells (**Figure 5D**). These data indicate that Abl kinase is involved at the step of clathrin-mediated endocytosis during HCV infection. Moreover, we provide data suggesting that Abl represents a new host factor for HCV entry.

## Discussion

The HCV life cycle is highly dependent on cellular factors for viral entry, replication, assembly, and virion release. Abl, non-receptor tyrosine kinase, is a master regulator of multiple cellular processes controlling actin dynamics, proliferation, and differentiation. Previous studies have demonstrated that Abl tyrosine kinase has been involved in entry of many viruses. Coxsackievirus triggers Abl and Fyn kinase activation to permit entry by movement of virus particle to the tight junctions (Coyne and Bergelson, 2006). HIV-1 also exploits Abl kinase activity to promote F-actin rearrangement by stimulating Wave2 signaling at a post-hemifusion step (Harmon et al., 2010). Ebola virus usurps Abl kinase to phosphorylate tyrosine 13 of VP40 for its own replication (Garcia et al., 2012). Moreover, dengue-virus entry is blocked by small-molecule inhibitors including Abl kinase (Schmidt et al., 2012).

In the present study, we showed that knockdown of Abl impaired HCV propagation. We also demonstrated that HCV propagation was significantly disrupted by either imatinib or dasatinib, and inhibition occurred in a dose-dependent manner. Moreover, these Abl kinase inhibitors exerted no cellular cytotoxicity to Huh7.5 cells. These data indicate that Abl tyrosine kinase is required for HCV propagation. Imatinib and dasatinib are well-known drugs for Philadelphia chromosome-positive chronic myeloid leukemia (CML) to inhibit the activity of Bcr-Abl, a tyrosine kinase of Bcr and Abl fusion protein. Imatinib impairs Abl kinase activity by binding a distinctive inactive conformation of the activation loop of Abl (Schindler et al., 2000), and dasatinib blocks Abl activation by binding an active conformation of kinase domain of Abl (Weisberg et al., 2007). We next determined which step of the HCV life cycle required cellular Abl. We demonstrated that siRNA-mediated knockdown of Abl displayed no effect on HCV replication in both HCV replicon and HCV-infected cells. We further verified that chemical inhibition of Abl kinase had no effect on HCV replication, indicating that Abl was not involved in the replication step of the HCV life cycle. We therefore explored the possible involvement of Abl in HCV entry. Using HCVpp entry assay, we demonstrated that silencing of Abl expression impaired HCVpp entry of both JFH1 and H77 strains. Moreover, time-of-addition assays verified that Abl kinase activity was specifically involved in HCV entry.

Hepatitis C virus enters cells via clathrin-mediated endocytosis and fuses with the host membrane in the late endosome. Bafilomycin A1, an inhibitor of vacuolar H^+^-ATPase, impairs vesicle acidification and thus inhibits HCV infection. Similar to bafilomycin A1, imatinib also inhibited HCV infection at the entry step of the HCV life cycle. It has been previously reported that phenothiazine intercalates into cholesterol-rich domain of the target membrane and increases membrane fluidity. Thus phenothiazine inhibits HCV entry at the fusion step of virus and host cells (Chamoun-Emanuelli et al., 2013). Trifluoperazine, a moiety of phenothiazine, significantly reduced membrane fluidity, whereas imatinib displayed no effect on membrane fluidity. These data indicate that Abl kinase has no functional involvement in membrane fluidity.

We next explored the possible involvement of Abl in clathrin-mediated endocytosis of HCV. Clathrin-mediated endocytosis of transferrin is a constitutive process of trophic iron uptake. We therefore analyzed the effect of imatinib treatment on transferrin uptake. As reported previously (Cao et al., 2016), chlorpromazine, a blocker for clathrin-dependent endocytosis, efficiently blocked transferrin uptake (**Figure 5B**). Here, we showed that imatinib treatment caused a significant reduction in transferrin uptake. This result indicates that Abl is involved in clathrin-mediated endocytosis. We further demonstrated that HCV-induced clathrin aggregation was inhibited by either chemical inhibition or siRNA-mediated silencing of Abl kinase. All these data indicate that Abl regulates HCV entry via clathrin-mediated endocytosis.

Since knockdown of Abl impaired Abl kinase activity in HCV-infected cells, we wondered whether Abl kinase was activated by HCV. Surprisingly, Abl kinase activity was markedly increased at 10 min after Jc1 infection as compared with mock infection. Likewise, Abl kinase activity was markedly increased at 10 min after sE2 treatment as compared with mock treatment, suggesting that Abl may be activated via protein interplay between E2 and host entry factor. These data, together with time of addition assay, suggest that Abl may be activated after binding of HCV envelope to host CD81 to facilitate clathrin-mediated endocytosis. Taken together, HCV modulates Abl activity for viral propagation and thus Abl may be a host target for therapeutic intervention.

## Author Contributions

All authors have given approval to the final version of the manuscript. SM performed experiments, analyzed data, and wrote the manuscript; Y-SL, DS, CP performed experiments; J-BP, SK, MW provided reagents and analyzed data; SH designed experiments, supervised the study, and wrote the manuscript.

## Conflict of Interest Statement

The authors declare that the research was conducted in the absence of any commercial or financial relationships that could be construed as a potential conflict of interest.

## Abbreviations

DAAs: direct-acting antivirals
HCV: hepatitis C virus
HCVcc: cell culture derived HCV
HTAs: host-targeted antivirals.

## References

[B1] Backert S. Feller S. M. Wessler S. (2008). Emerging roles of Abl family tyrosine kinases in microbial pathogenesis. *Trends Biochem. Sci.* 33 80–90. 10.1016/j.tibs.2007.10.006 18182299

[B2] Baldick C. J. Wichroski M. J. Pendri A. Walsh A. W. Fang J. Mazzucco C. (2010). A novel small molecule inhibitor of hepatitis C virus entry. *PLoS Pathog.* 6:e1001086 10.1371/journal.ppat.1001086 PMC2936744 20838466

[B3] Bartosch B. Vitelli A. Granier C. Goujon C. Dubuisson J. Pascale S. (2003). Hepatitis C virus E2 links soluble human CD81 and SR-B1 protein. *J. Biol. Chem.* 278 41624–41630. 10.1074/jbc.M305289200 12913001

[B4] Blanchard E. Belouzard S. Goueslain L. Wakita T. Dubuisson J. Wychowski C. (2006). Hepatitis C virus entry depends on clathrin-mediated endocytosis. *J. Virol.* 80 6964–6972. 10.1128/JVI.00024-06 16809302 PMC1489042

[B5] Brazzoli M. Bianchi A. Filippini S. Weiner A. Zhu Q. Pizza M. (2008). CD81 is a central regulator of cellular events required for hepatitis C virus infection of human hepatocytes. *J. Virol.* 82 8316–8329. 10.1128/JVI.00665-08 18579606 PMC2519688

[B6] Bush C. O. Pokrovskii M. V. Saito R. Morganelli P. Canales E. Clarke M. O. (2014). A small-molecule inhibitor of hepatitis C virus infectivity. *Antimicrob. Agents Chemother.* 58 386–396. 10.1128/AAC.02083-13 24165192 PMC3910743

[B7] Cao H. Schroeder B. Chen J. Schott M. B. McNiven M. A. (2016). The endocytic fate of the transferrin receptor is regulated by c-Abl kinase. *J. Biol. Chem.* 291 16424–16437. 10.1074/jbc.M116.724997 27226592 PMC4974358

[B8] Carlson A. Gregorich Z. Striker R. (2013). Telaprevir to boceprevir switch highlights lack of cross-reactivity. *Clin. Infect. Dis.* 56 552–554. 10.1093/cid/cis960 23166191 PMC3732052

[B9] Chamoun-Emanuelli A. M. Peacheur E. I. Simeon R. L. Huang D. Cremer P. S. Chen Z. (2013). Phenothiazines inhibit hepatitis C virus entry, likely by increasing the fluidity of cholesterol-rich membranes. *Antimicrob. Agents Chemother.* 57 2571–2581. 10.1128/AAC.02593-12 23529728 PMC3716126

[B10] Colicelli J. (2010). ABL tyrosine kinases: evolution of function, regulation, and specificity. *Sci. Signal.* 3:re6 10.1126/scisignal.3139re6 PMC2954126 20841568

[B11] Coyne C. B. Bergelson J. M. (2006). Virus-induced Abl and Fyn kinase signals permit coxsackievirus entry through epithelial tight junctions. *Cell* 124 119–131. 10.1016/j.cell.2005.10.035 16413486

[B12] Farquhar M. J. Hu K. Harris H. J. Davis C. Brimacombe C. L. Fletcher S. J. (2012). Hepatitis C virus induces CD81 and claudin-1 endocytosis. *J. Virol.* 86 4305–4316. 10.1128/JVI.06996-11 22318146 PMC3318669

[B13] Garcia M. Cooper A. Shi W. Bornmann W. Carrion R. Kalman D. (2012). Productive replication of Ebola virus is regulated by the c-Abl1 tyrosine kinase. *Sci. Transl. Med.* 4 123ra124. 10.1126/scitranslmed.3003500 PMC4794994 22378924

[B14] Giannini C. Brechot C. (2003). Hepatitis C virus biology. *Cell Death Differ.* 10(Suppl. 1), S27–S38. 10.1038/sj.cdd.4401121 12655344

[B15] Harmon B. Campbell N. Ratner L. (2010). Role of Abl kinase and the Wave2 signaling complex in HIV-1 entry at a post-hemifusion step. *PLoS Pathog.* 6:e1000956 10.1371/journal.ppat.1000956 PMC2887473 20585556

[B16] Lee M. Yang J. Jo E. Lee J. Y. Kim H. Y. Bartenschlager R. (2017). A novel inhibitor IDPP interferes with entry and egress of HCV by targeting glycoprotein E1 in a genotype-specific manner. *Sci. Rep.* 7:44676 10.1038/srep44676 PMC5363083 28333153

[B17] Lindenbach B. D. Rice C. M. (2005). Unravelling hepatitis C virus replication from genome to function. *Nature* 436 933–938. 10.1038/nature04077 16107832

[B18] Lindenbach B. D. Rice C. M. (2013). The ins and outs of hepatitis C virus entry and assembly. *Nat. Rev. Microbiol.* 11 688–700. 10.1038/nrmicro3098 24018384 PMC3897199

[B19] Liu S. Yang W. Shen L. Turner J. R. Coyne C. B. Wang T. (2009). Tight junction proteins claudin-1 and occludin control hepatitis C virus entry and are downregulated during infection to prevent superinfection. *J. Virol.* 83 2011–2014. 10.1128/JVI.01888-08 19052094 PMC2643775

[B20] Lupberger J. Zeisel M. B. Xiao F. Thumann C. Fofana I. Zona L. (2011). EGFR and EphA2 are host factors for hepatitis C virus entry and possible targets for antiviral therapy. *Nat. Med.* 17 589–595. 10.1038/nm.2341 21516087 PMC3938446

[B21] Monazahian M. Böhme I. Bonk S. Koch A. Scholz C. Grethe S. (1999). Low density lipoprotein receptor as a candidate receptor for hepatitis C virus. *J. Med. Virol.* 57 223–229. 10.1002/(SICI)1096-9071(199903)57:3<223::AID-JMV2>3.0.CO;2-4 10022791

[B22] Murray J. C. Aldeghaither D. Wang S. Nasto R. E. Jablonski S. A. Tang Y. (2014). c-Abl modulates tumor cell sensitivity to antibody-dependent cellular cytotoxicity. *Cancer Immunol. Res.* 2 1186–1198. 10.1158/2326-6066.CIR-14-0083 25300860 PMC4258447

[B23] Ngo H. T. Pham L. V. Kim J. W. Lim Y. S. Hwang S. B. (2013). Modulation of mitogen-activated protein kinase-activated protein kinase 3 by hepatitis C virus core protein. *J. Virol.* 87 5718–5731. 10.1128/JVI.03353-12 23487458 PMC3648169

[B24] Nguyen L. N. Lim Y. S. Pham L. V. Shin H. Y. Kim Y. S. Hwang S. B. (2014). Stearoyl-CoA desaturase 1 is associated with hepatitis C virus replication complex and regulates viral replication. *J. Virol.* 88 12311–12325. 10.1128/JVI.01678-14 25122791 PMC4248942

[B25] Parasassi T. De Stasio G. d’Ubaldo A. Gratton E. (1990). Phase fluctuation in phospholipid membranes revealed by Laurdan fluorescence. *Biophys. J.* 57 1179–1186. 10.1016/S0006-3495(90)82637-0 2393703 PMC1280828

[B26] Park C. Min S. Park E. M. Lim Y. S. Kang S. Suzuki T. (2015). Pim kinase interacts with nonstructural 5A protein and regulates hepatitis C virus entry. *J. Virol.* 89 10073–10086. 10.1128/JVI.01707-15 26202252 PMC4577879

[B27] Pham T. M. Tran S. C. Lim Y. S. Hwang S. B. (2017). Hepatitis C virus-induced Rab32 aggregation and its implications for virion assembly. *J. Virol.* 91:e01662-16. 10.1128/JVI.01662-16 PMC5244344 27852857

[B28] Pietschmann T. Kaul A. Koutsoudakis G. Shavinskaya A. Kallis S. Steinmann E. (2006). Construction and characterization of infectious intragenotypic and intergenotypic hepatitis C virus chimeras. *Proc. Natl. Acad. Sci. U.S.A.* 103 7408–7413. 10.1073/pnas.0504877103 16651538 PMC1455439

[B29] Reddy E. P. Smith M. J. Srinivasan A. (1983). Nucleotide sequence of Abelson murine leukemia virus genome: structural similarity of its transforming gene product to other onc gene products with tyrosine-specific kinase activity. *Proc. Natl. Acad. Sci. U.S.A.* 80 3623–3627. 10.1073/pnas.80.12.3623 6304726 PMC394102

[B30] Saito I. Miyamura T. Ohbayashi A. Harada H. Katayama T. Kikuchi S. (1990). Hepatitis C virus infection is associated with the development of hepatocellular carcinoma. *Proc. Natl. Acad. Sci. U.S.A.* 87 6547–6549. 10.1073/pnas.87.17.6547 2168552 PMC54573

[B31] Sato M. Maruoka M. Takeya T. (2012). Functional mechanisms and roles of adaptor proteins in abl-regulated cytoskeletal actin dynamics. *J. Signal Transduct.* 2012:414913 10.1155/2012/414913 PMC3362954 22675626

[B32] Scarselli E. Ansuini H. Cerino R. Roccasecca R. M. Acali S. Filocamo G. (2002). The human scavenger receptor class B type I is a novel candidate receptor for the hepatitis C virus. *EMBO J.* 21 5017–5025. 10.1093/emboj/cdf529 12356718 PMC129051

[B33] Schindler T. Bornmann W. Pellicena P. Miller W. T. Clarkson B. Kuriyan J. (2000). Structural mechanism for STI-571 inhibition of abelson tyrosine kinase. *Science* 289 1938–1942. 10.1126/science.289.5486.1938 10988075

[B34] Schmidt A. G. Lee K. Yang P. L. Harrison S. C. (2012). Small-molecule inhibitors of Dengue-virus entry. *PLoS Pathog.* 8:e1002627 10.1371/journal.ppat.1002627 PMC3320583 22496653

[B35] Ten Hoeve J. Arlinghaus R. B. Guo J. Q. Heisterkamp N. Groffen J. (1994). Tyrosine phosphorylation of CRKL in Philadelphia+ leukemia. *Blood* 84 1731–1736. 7521685

[B36] Weisberg E. Manley P. W. Cowan-Jacob S. W. Hochhaus A. Griffin J. D. (2007). Second generation inhibitors of BCR-ABL for the treatment of imatinib-resistant chronic myeloid leukaemia. *Nat. Rev. Cancer* 7 345–356. 10.1038/nrc2126 17457302

[B37] Woodring P. J. Hunter T. Wang J. Y. (2003). Regulation of F-actin-dependent processes by the Abl family of tyrosine kinases. *J. Cell Sci.* 116 2613–2626. 10.1242/jcs.00622 12775773

[B38] Xu J. Millard M. Ren X. Cox O. T. Erdreich-Epstein A. (2010). c-Abl mediates endothelial apoptosis induced by inhibition of integrins αvβ3 and αvβ5 and by disruption of actin. *Blood* 115 2709–2718. 10.1182/blood-2009-05-223776 20124512 PMC2852370

[B39] Yamane D. McGivern D. R. Wauthier E. Yi M. Madden V. J. Welsch C. (2014). Regulation of the hepatitis C virus RNA replicase by endogenous lipid peroxidation. *Nat. Med.* 20 927–935. 10.1038/nm.3610 25064127 PMC4126843

[B40] Zona L. Lupberger J. Sidahmed-Adrar N. Thumann C. Harris H. J. Barnes A. (2013). HRas signal transduction promotes hepatitis C virus cell entry by triggering assembly of the host tetraspanin receptor complex. *Cell Host Microbe* 13 302–313. 10.1016/j.chom.2013.02.006 23498955

